# Clinical Equivalence of Trubond® and Ethibond® Braided Polyester Sutures for Valvular Prosthesis Fixation During Aortic or Mitral Valve Replacement: A Single-Blind Randomized Controlled Trial

**DOI:** 10.7759/cureus.41117

**Published:** 2023-06-28

**Authors:** Neelam B Desai, P.S. Seetharama Bhat, Chandra Sena M, Siddharth S, CH Praveen, Ashok K Moharana, Deepak TS

**Affiliations:** 1 Cardio Vascular and Thoracic Surgery, Sri Sathya Sai Institute of Higher Medical Sciences, Puttaparthi, IND; 2 Cardio Vascular and Thoracic Surgery, Sri Jayadeva Institute of Cardiovascular Sciences and Research, Bengaluru, IND; 3 Clinical Affairs, Healthium Medtech Limited, Bengaluru, IND

**Keywords:** valvular prosthesis, polyester suture, paravalvular leak, mitral valve, aortic valve

## Abstract

Background

Paravalvular leak (PVL) following valve replacement is a serious cardiovascular complication that increases morbidity and mortality. Valve replacement with the interrupted suture technique using polyester suture provides adequate tensile strength and reduces the probability of tissue reaction. This study compared the clinical equivalence of Trubond®(Healthium) and Ethibond® (Ethicon, Johnson & Johnson) braided polyester sutures for valvular prosthesis fixation using interrupted suturing, with respect to the proportion of subjects having PVL after aortic valve (AV) or mitral valve (MV) replacement.

Methodology

Patients undergoing AV/MV replacement were enrolled and randomized in this study. The primary endpoint of this prospective, multicentric, two-arm, randomized (1:1), parallel-group, single-blind study (December 2020-October 2022) was the presence of PVL in Trubond® (n = 40) and Ethibond® (n = 42) groups within 26 weeks of surgery. The secondary endpoints included event rate of all-cause mortality, cardiac death, stroke, myocardial infarction, re-hospitalization, re-intervention, wound infection, operative time, intraoperative suture parameters, postoperative hospital stay, time to resume normal activities and work, quality of life, patient satisfaction, and adverse events in both groups.

Results

Patients who underwent AV/MV replacement and were followed up until 26 weeks had no incidence of PVL or other postoperative complications. No requirement for readmission or re-intervention was noted in both groups. Intraoperative suture handling characteristics, operative time, and hospital stay were also comparable between the groups. With each follow-up, subjects in both groups exhibited improved postoperative functional abilities, quality of life, and health status.

Conclusions

Trubond® braided polyester suture is clinically equivalent to Ethibond® braided polyester suture. Trubond® suture is safe and effective for valvular prosthesis fixation in patients undergoing AV or MV replacement.

## Introduction

One of the increasing causes of cardiovascular morbidity and mortality is valvular heart disease (VHD) [1]. The Global Burden of Disease study involving 195 countries found that 12.6 and 18.1 million people were affected by the disease of the calcific aortic valve (AV) and degenerative mitral valve (MV), respectively, between 1990 and 2017 [2]. The prevalence of AV stenotic disease is higher among developed countries, while rheumatic heart disease is more frequent in developing countries, particularly in the Indian subcontinent [3]. An Indian study reported more than 90% of cases (<60 years) of VHD that included 29.6% cases of mitral stenosis and 34.5% cases of combined MV and AV disease [4]. Duration of the valve lesions and their severity can have a secondary impact on other non-pathological valves. Correcting the pathological valve may alter the severity of the lesion in multivalvular heart disease [5].

Treatment of VHD involves repair of the native valve or replacement of the valve with mechanical or biological prostheses [6] that restore unidirectional blood flow (transvalvular flow) [7]. However, following valve replacement, serious complications such as paravalvular leak (PVL) may emerge that hampers the unidirectional flow of blood across the cardiac chambers [8,9]. Approximately 2-5% of PVL cases cause clinically relevant symptoms of hemolytic anemia, congestive heart failure, or death due to sepsis [8], and <2% require re-operation [10].

The interrupted suture technique for MV replacement results in a lower incidence of peri-prosthetic leakage [11]. For AV replacement, the technique has similar effectiveness and allows the implantation of larger prostheses by providing a larger effective orifice area [12]. Furthermore, the polyester suture is considered safe and effective because of its inherent elasticity and adequate tensile strength, as well as the ability to prevent dehiscence, surgical site infection (SSI), and bleeding [13]. Many previous studies have used polypropylene mattress sutures [14], pledged sutures [12,15], and polyester sutures [12] for valve replacement surgery. However, the clinical equivalence of two commonly used brands of braided polyester sutures, Trubond® (Healthium) and Ethibond® (Ethicon, Johnson & Johnson), with respect to the development of PVL within 26 weeks of valve replacement surgery is not established. Therefore, the present study compared Trubond® and Ethibond® sutures for interrupted suturing of the valvular prosthesis in patients undergoing AV or MV replacement.

## Materials and methods

Study design

This prospective, multicentric, randomized, two-arm, parallel-group, single-blind study was conducted between December 2020 and October 2022. The primary study objective was the assessment of the event rate of PVL in Trubond® and Ethibond® groups within 26 weeks of index surgery. Secondary objectives included assessment of major adverse events (all-cause mortality, cardiac death, stroke, myocardial infarction, and surgical re-intervention), infection, overall intraoperative handling, time taken to resume normal activities and work, tissue reaction, material problems, adverse events, postoperative discomfort, and overall patient satisfaction score.

Ethical approval

Ethics committee approval from both trial sites was obtained before initiating trial activity. The trial was prospectively registered in the Clinical Trial Registry of India (CTRI/2020/04/024608).

Study participants

Adult patients aged ≥18 or <70 years requiring AV or MV prosthesis implantation through open heart surgery were eligible to participate in the study after providing written informed consent.

Individuals who required multivalve surgery, had implanted pacemakers or implantable cardiac defibrillators, or had a history of coronary artery bypass graft or any valve surgery, bleeding disorders, or polyester allergy were excluded. Individuals, with the habit of drug abuse or experimental drug or medical device administration within 30 days of the study and females who were pregnant or had childbearing potential with positive urine pregnancy tests were excluded. Participants with mental disorders, learning disabilities, and language barriers and those who were participating in another surgical study or were unlikely to comply with surgical procedures or study visits were also excluded.

Study setting

The study was conducted at (i) the Department of Cardio-Thoracic and Vascular Surgery, Sri Sathya Sai Institute of Higher Medical Sciences, Andhra Pradesh, India, and (ii) the Department of Cardio-Thoracic Surgery, Sri Jayadeva Institute of Cardiovascular Sciences and Research, Karnataka, India.

Intervention

Trubond® (Healthium Medtech Ltd) and Ethibond® (Ethicon, Johnson & Johnson) are non-absorbable, braided, sterile, surgical sutures prepared from the fine filaments of polyethylene terephthalate. Both sutures are indicated for general soft tissue approximation and/or ligation during ophthalmic, cardiovascular, or neurological procedures.

Study procedure

All study participants underwent midline sternotomy followed by cardiopulmonary bypass. After arresting the heart with cardioplegia, MV or AV was replaced. Prostheses (mechanical or biological) were secured to the annulus with an interrupted polyester suture technique. For MV replacement, the pledged polyester suture was passed through the sewing ring of the valvular prosthesis for fixation. For AV replacement, the suture was placed into the annulus and through the skirt of the valve. Pledgets were used only if the aortic annulus was dilated.

The study examined outcomes over a course of 26 weeks consisting of a screening visit (week 26 to day 1), baseline visit (day of surgery or day 0), day 3, day 4-15 (day of discharge or DOD), and follow-up visits at week 6 post-DOD, week 12 post-DOD, and week 24 post-DOD.

Baseline characteristics

Baseline demographics (age, gender, ethnicity, weight, height, body mass index, family history of cardiovascular disease, reason for AV/MV replacement, alcohol and smoking history, and American Society of Anesthesiologists (ASA) classification), vital signs (pulse rate and systolic and diastolic blood pressure), functional capacity/ability (total distance walked in six minutes and New York Heart Association (NYHA) classification), medical/surgical history, physical examination for any serious problem, the reason for valve replacement, and echocardiographic characteristics (ejection fraction, mean transvalvular gradient, grading severity of regurgitation, and presence of PVL) were assessed. Valve replacement surgery was indicated based on the diagnosis of symptomatic valvular stenosis or regurgitation. The patient’s preoperative echocardiographic findings were used to corroborate the diagnosis.

Study outcomes

Primary Endpoint

The primary endpoint was the proportion of patients having PVL within 26 weeks of index surgery.

Secondary Endpoints

Event rate of postoperative complications, viz. all-cause mortality, cardiac death, stroke, myocardial infarction, superficial/deep incisional SSI, re-hospitalization, and re-intervention, was recorded on days 0, 3, and DOD, and on weeks 6, 12, and 24 post-DOD. Intraoperative suture parameters, viz. ease of passage through tissue, first-throw knot holding, knot security, knot tie-down smoothness, stretch capacity, memory, suture fraying, and tissue drag, were assessed as 1 (poor), 2 (fair), 3 (good), 4 (very good), and 5 (excellent). Intraoperative antibiotic prophylaxis, thrombosis prophylaxis, size of sutures, number of sutures used, blood transfusions, operative time, cardiopulmonary bypass time, and aortic cross-clamp time were also recorded. Postoperative echocardiographic characteristics (ejection fraction, mean transvalvular gradient, grading severity of regurgitation, and presence of PVL on DOD and week 24 post-DOD), length of postoperative intensive care unit (ICU)/hospital stay, duration of the drain, functional capacity, and time to resume normal activities and work were noted.

In addition, quality of life (QoL) was evaluated based on the EuroQoL five-dimensional (mobility, self-care, usual activities, pain/discomfort, and anxiety/depression) three-level (no problems, some problems, and extreme problems) questionnaire or the EQ-5D-3L. A global assessment of the patient’s health was assessed based on values ranging between 0 (worst imaginable health) and 100 (best imaginable health) on the EuroQoL-Visual Analog Scale (EQ-VAS). Both QoL and the patient’s health were recorded at screening, DOD, and weeks 6, 12, and 24 post-DOD.

In addition to the study endpoints, the occurrence of any untoward medical or clinical sign, disease, or injury during the study period was recorded as an adverse event. The concomitant or prescribed medication details were also captured.

Sample size

In 1978, Beddermann et al. reported a 5% cumulative proportion of PVL for both AV and MV, positioned with interrupted sutures [16]. The findings were considered for sample size calculation of the Ethibond® arm. In the Trubond® arm, the cumulative proportion of the PVL was assumed to be 5.5%. A sample size of 74 (37 in each group) was calculated, with 5% type I error, 80% power, and 15% margin of non-inferiority. Adding 20% post-randomization exclusion/drop-out, the final sample size was 88 (n = 44 in each arm).

Randomization and blinding

Randomization numbers were generated in a 1:1 ratio using freely available software. The numbers were concealed using the sequentially numbered opaque sealed envelope technique. The patients were unaware of the allocation status, but the operating staff was not blinded.

Statistical analysis

Per-protocol or PP analysis set was used to present the patient’s complete data on the primary effectiveness parameter at 26 weeks follow-up, having no major protocol deviations. The chi-square test was used to compare qualitative data in the form of proportions or percentages. Both parametric and non-parametric continuous data were expressed as mean ± SD. According to the distribution of the data, the t-test or Mann-Whitney U test was used. For both qualitative and continuous variables, p < 0.05 was regarded as statistically significant. Primary data were compared using the chi-square test, and secondary endpoints were compared depending on the quantitative or qualitative nature of the variables. For analyzing the data, SPSS software version 28.0 (IBM Corp., Armonk, NY, USA) was used.

## Results

**Figure 1:**
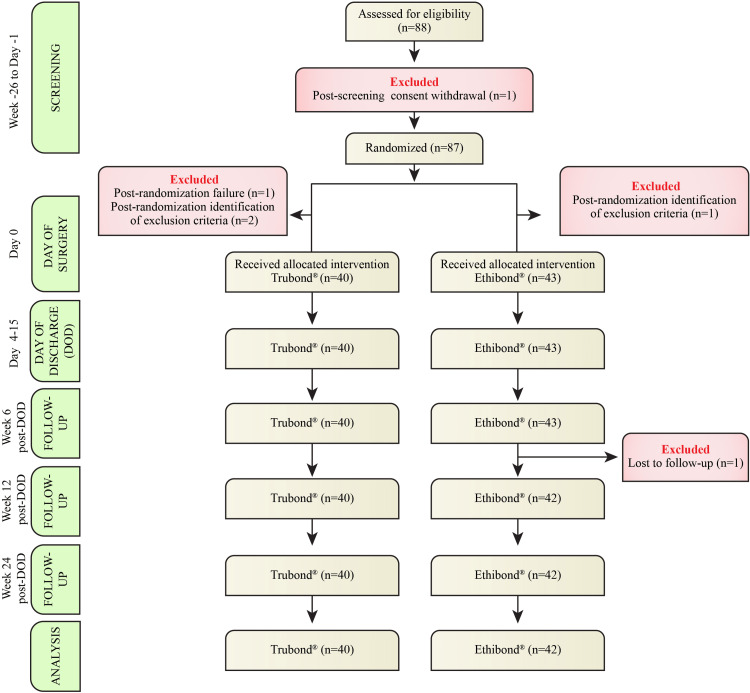
CONSORT flow chart of the study profile.

A total of 88 participants were screened for eligibility between December 2020 and March 2022; one participant withdrew consent after the screening. Due to post-randomization failure, post-randomization identification of exclusion criteria, and loss to follow-up, a total of five participants were excluded (Figure 1).

Finally, the PP analysis set consisted of 82 participants randomized to the Trubond® (n = 40) and Ethibond® (n = 42) groups who completed the trial.

Baseline characteristics

Baseline demographics, vital signs, ASA grade, and medical/surgical history were comparable between the groups (Table 1).

**Table 1:** Baseline characteristics of the study participants. AV: aortic valve; ASA: American Society of Anesthesiologists; BMI: body mass index; MV: mitral valve; SD: standard deviation

Subject characteristics	Trubond^®^ (n = 40)	Ethibond^®^ (n = 42)	P-value
Demographics, mean ± SD			
Age (years)	45.1 ± 9.6	41.8 ± 9.8	0.14
Weight (kg)	58.0 ± 10.9	55.1 ± 10. 7	0.22
Height (cm)	160.3 ± 8.8	157.9 ± 10.8	0.29
BMI (kg/m^2^)	22.7 ± 4.3	22.2 ± 4.3	0.62
Vital signs, mean ± SD			
Pulse rate (beats per minute)	82.1 ± 9.0	83.0 ± 11.1	0.69
Systolic blood pressure (mmHg)	115.7 ± 11.8	116.4 ± 12.2	0.79
Diastolic blood pressure (mmHg)	72.6 ± 8.7	75.2 ± 10.0	0.22
ASA grading, n (%)			
ASA I	16 (40.0)	21 (50.0)	0.65
ASA II	4 (10.0)	4 (9.5)	
ASA III	20 (50.0)	17 (40.5)	
Medical/surgical history, n (%)	30 (75.0)	29 (69.1)	0.77
Reason for AV replacement, n (%)			
Severe stenosis	2 (18.2)	1 (10.0)	0.57
Moderate stenosis	0	1 (10.0)	
Severe regurgitation	2 (18.2)	2 (20.0)	
Severe stenosis + mild regurgitation	4 (36.4)	5 (50.0)	
Severe stenosis + moderate regurgitation	2 (18.2)	0	
Moderate stenosis + severe regurgitation	1 (9.1)	0	
Mild stenosis + moderate regurgitation	0	1 (10.0)	
Reason for MV replacement, n (%)			
Severe stenosis	6 (20.7)	9 (28.1)	0.75
Severe regurgitation	1 (3.5)	1 (3.1)	
Severe stenosis + mild regurgitation	9 (31.0)	5 (15.6)	
Severe stenosis + moderate regurgitation	8 (27.6)	10 (31.3)	
Severe stenosis + severe regurgitation	1 (3.5)	1 (3.1)	
Moderate stenosis + severe regurgitation	3 (10.3)	1 (3.1)	
Moderate stenosis + moderate regurgitation	1 (3.5)	1 (3.1)	
Mild stenosis + severe regurgitation	0	3 (9.4)	
Mild stenosis + moderate regurgitation	0	1 (3.1)	

**Figure 2:**
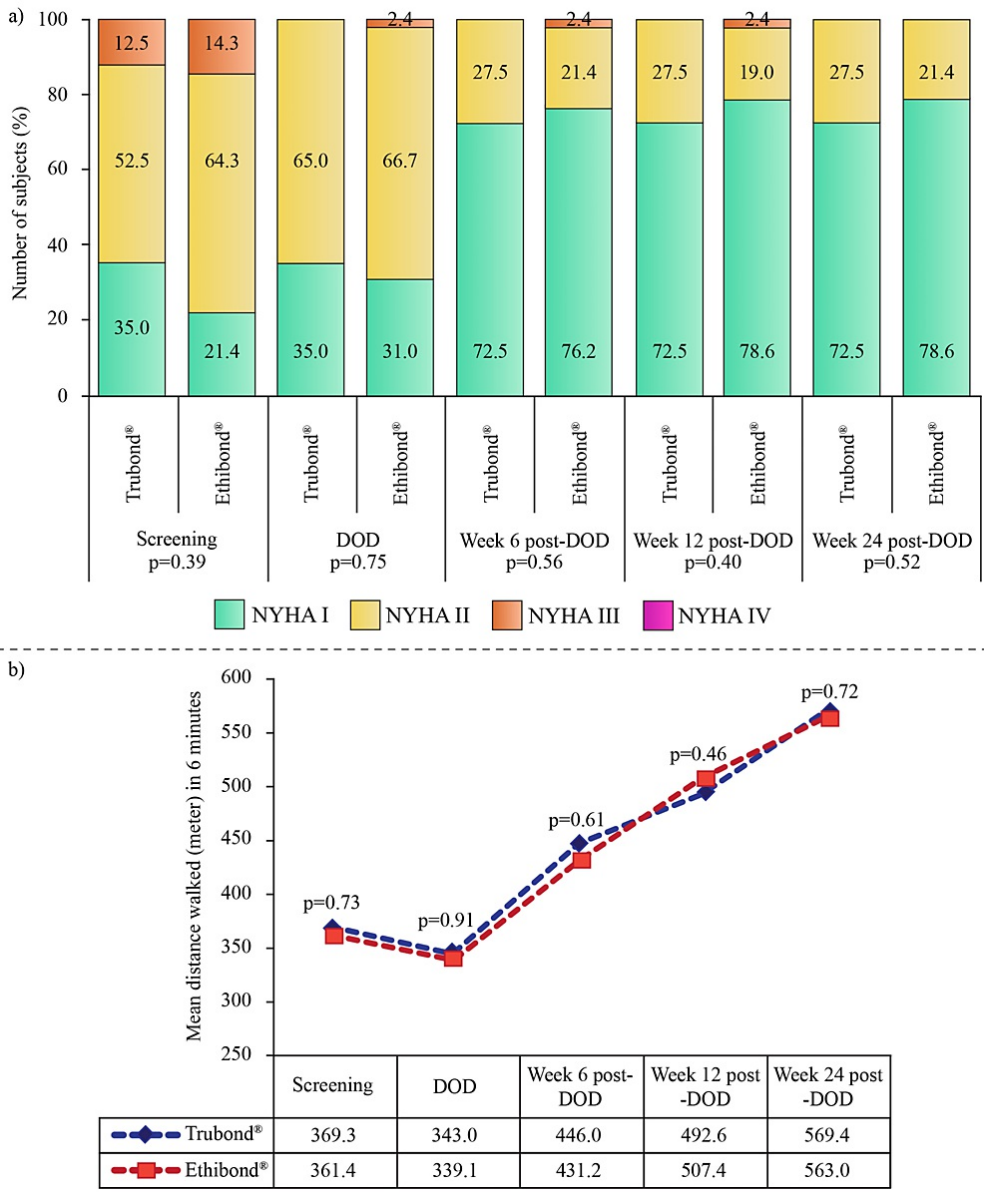
Comparison of (a) functional ability (NYHA classification) and (b) functional capacity (mean distance walked in six minutes) between the Trubond® (n = 40) and Ethibond® (n = 42) groups. DOD: day of discharge; NYHA: New York Heart Association

In the Trubond® group, 45.0% and in the Ethibond® group, 47.6% of subjects were males (p = 0.83). In the Trubond® and Ethibond® groups, 95.0% and 97.6% of subjects, respectively, were Indians; the rest were non-Indian Asians (p = 0.61). Both groups were comparable in terms of alcoholism (p = 0.35) and smoking history (p = 0.054). Physical examination revealed abnormal cardiovascular system (p = 1.00) in all subjects of both groups and pedal edema (p = 0.96) in one subject of each group. None of the subjects who participated in the study had a family history of cardiovascular disease. Before surgery, transthoracic echocardiography was performed in both groups (p = 1.00). Reasons for valve replacement are provided in Table 1. Severe stenosis along with mild regurgitation was the reason for AV replacement in the majority of subjects in both Trubond® (36.4%) and Ethibond® (50.0%) groups. The frequent reasons for MV replacement were severe stenosis along with mild regurgitation (31.0%), followed by severe stenosis and moderate regurgitation (27.6%) in the Trubond® group, and severe stenosis along with moderate regurgitation (31.3%) and severe stenosis alone (28.1%) in the Ethibond® group. The preoperative functional capacity/ability was comparable between the groups (Figure 2).

Primary endpoint analysis

The study participants were evaluated for PVL at all postoperative follow-ups. There was no incidence of PVL of mild, moderate, or severe grade among the participants of both groups.

Secondary endpoint analysis

**Figure 3:**
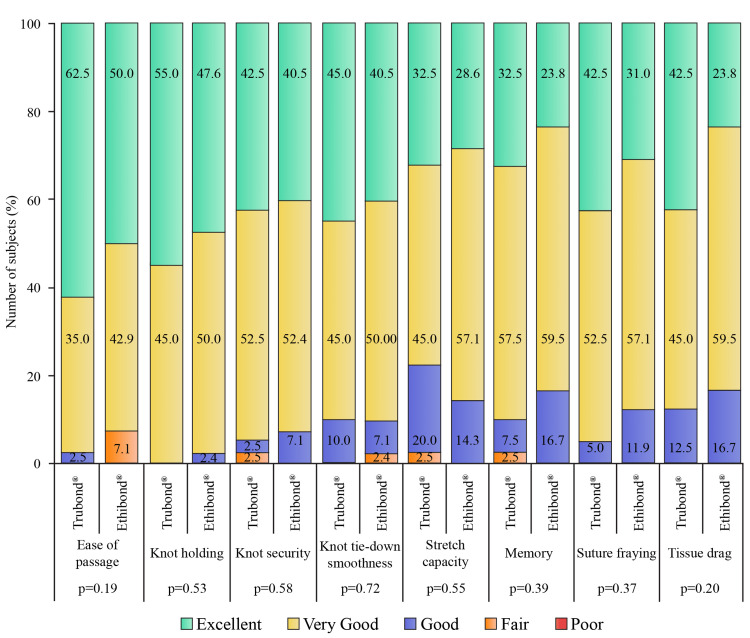
Intraoperative suture handling characteristics of the Trubond® (n = 40) and Ethibond® (n = 42) groups.

Antibiotic prophylaxis was used in all subjects. AV and MV replacement was performed in 11 (27.5%) and 29 (72.5%) subjects of the Trubond® group, respectively, and 10 (23.8%) and 32 (76.2%) subjects of the Ethibond® group, respectively (p = 0.70). In the Trubond® and Ethibond® groups, mechanical bileaflet (35.0 vs. 38.1%), mechanical tilting disc (55.0 vs. 57.1%), bovine bioprosthesis (2.5 vs. 0%), and porcine bioprosthesis (7.5 vs. 4.8%) valves were used (p = 0.96). Size No. 2 Trubond® or Ethibond® braided polyester suture was applied. The results of intraoperative handling characteristics (ease of passage, knot holding, knot security, knot tie-down, stretch capacity, memory, suture fraying, and tissue drag) of both sutures were comparable (Figure 3).

None of the suture characteristics scored poor, and no suture-related challenges were recorded. However, the number of sutures used showed a significant difference (p = 0.003) between the groups, which is subjective to annulus size (Table 2).

**Table 2:** Intraoperative and postoperative profile of the study participants. DOD: day of discharge; EQ-5D: EuroQoL five-dimensional questionnaire; ICU: intensive care unit; SD: Standard deviation; *: p < 0.05

Subject profile	Trubond^®^ (n = 40)	Ethibond^®^ (n = 42)	P-value
Intraoperative, mean ± SD			
Number of sutures used	17.8 ± 3.5	15.6 ± 3.0	0.003^*^
Number of blood transfusions	0.4 ± 0.6	0.5 ± 1.0	0.64
Total operative time (hours)	3.7 ± 2.4	3.3 ± 1.5	0.41
Cardiopulmonary bypass time (minutes)	133.1 ± 33.8	137.1 ± 29.4	0.56
Aortic cross-clamp time (minutes)	103.0 ± 31.9	111.1 ± 32.4	0.26
Postoperative, mean ± SD			
Duration of drain (days)	2.8 ± 2.0	3.9 ± 4.5	0.17
ICU stay duration (days)	4.5 ± 1.6	4.9 ± 1.9	0.33
Hospital stay duration (days)	7.3 ± 3.8	7.5 ± 4.1	0.80
Time to return to normal day-to-day activities (days)	15.7 ± 6.9	16.2 ± 6.8	0.71
Time to return to work (days)	24.8 ± 7.8	27.5 ± 7.2	0.10
EQ-5D, mean ± SD			
Time to achieve no pain/discomfort (days)	106.1 ± 57.4 (n = 35)	88.7 ± 58.7 (n=35)	0.22
Time to achieve no anxiety/depression (days)	36.9 ± 47.5 (n = 32)	34.7 ± 5.7 (n = 37)	0.85

Comparable numbers of pledget reinforcement were applied in the Trubond® (77.5%) and Ethibond® (90.5%) groups (p = 0.13). Thrombosis prophylaxis (acenocoumarol, 52.5 vs. 50.0%; clexane, 0 vs. 2.4%; heparin, 47.5 vs. 47.6%) was used in both the Trubond® and Ethibond® groups (p = 0.59). The other intraoperative characteristics are presented in Table 2. Good outcomes of surgery along with no peri-operative complications were noted in both groups (p = 1.00).

Pre and postoperative NYHA classifications are shown in Figure 2a. Starting from DOD, the number of subjects with NYHA I grade increased in both groups. Likewise, increasing mean functional capacities, recorded by a six-minute walk test (Figure 2b) were found in both groups. Surgical re-intervention, repeated procedure, and re-admission to the hospital were not required in any subject. During follow-up, no postoperative complications in terms of all-cause mortality, cardiac death, stroke, myocardial infarction, or superficial/deep incisional SSI were recorded. Drains were removed after the surgery depending on the output; the mean duration of the drain was comparable between the groups (Table 2). The other postoperative details are provided in Table 2.

Echocardiographic findings indicated little improvement in ejection fraction 26 weeks postoperatively. Pre and postoperative (DOD and week 24 post-DOD) echocardiographic (transthoracic) characteristics are summarized in Table 3.

**Table 3:** Preoperative and postoperative echocardiographic characteristics of the study participants. AV: aortic valve; MV: mitral valve; PVL: paravalvular leak; SD: standard deviation; α: preoperative; β: day of discharge; γ: week 24 post-day of discharge; #: missing data

Echocardiographic characteristics	^α^Trubond^®^ (n = 40)	^α^Ethibond^®^ (n = 42)	^α^P-value	^β^Trubond^®^ (n=40)	^β^Ethibond^®^ (n=42)	^β^P-value	^γ^Trubond^®^ (n=40)	^γ^Ethibond^®^ (n=42)	^γ^P-value
Ejection fraction (%), mean ± SD	55.9 ± 6.2	56.4 ± 8.8	0.80	56.7 ± 5.0	54.5 ± 5.8	0.07	57.6 ± 4.2	57.7 ± 3.9	0.95
AV mean transvalvular gradient (mmHg), mean ± SD	50. 6 ± 25.4 (n = 9)^#^	47.8 ± 37.8 (n=10)	0.86	15.4 ± 5.0 (n = 11)	11.5 ± 4.4 (n = 10)	0.08	14.6 ± 4.0 (n = 11)	12.6 ± 5.8 (n = 10)	0.38
MV mean transvalvular gradient (mmHg), mean ± SD	12.7 ± 5.8 (n = 29)	12.0 ± 4.0 (n = 31)^#^	0.56	3.9 ± 1.5 (n = 29)	3.7 ± 1.4 (n = 31)^#^	0.63	4.5 ± 1.3 (n = 26)^#^	4.0 ± 1.1 (n = 31)^#^	0.13
PVL, n (%)	0	0	-	0	0	-	0	0	-

Mild mitral valvular regurgitation on DOD was seen in only one (2.4%) subject in the Ethibond® group (p = 0.99). However, at the last follow-up, mild aortic valvular regurgitation was recorded in one (2.5%) subject in the Trubond® and two (4.8%) subjects in the Ethibond® group, while two (4.8%) mild mitral valvular regurgitation was noted in the Ethibond® group (p = 0.18).

**Figure 4:**
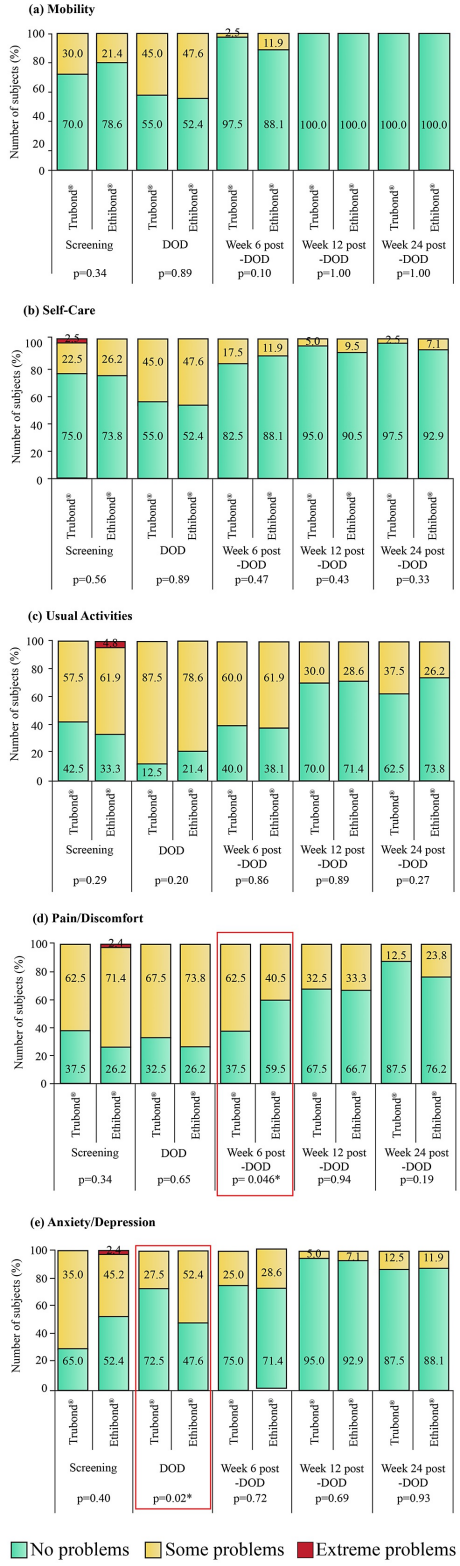
EQ-5D responses in the Trubond® (n = 40) and Ethibond® (n = 42) groups. EQ-5D: EuroQoL five-dimensional questionnaire; DOD: day of discharge; *: p < 0.05

At screening, the number of subjects with no problems (level 1), some problems (level 2), and extreme problems (level 3) for the five dimensions of the EQ-5D were comparable between the groups. An increasing number of subjects with no problems in mobility, self-care, usual activities, pain/discomfort, and anxiety/depression was recorded with each follow-up (Figure 4).

**Figure 5:**
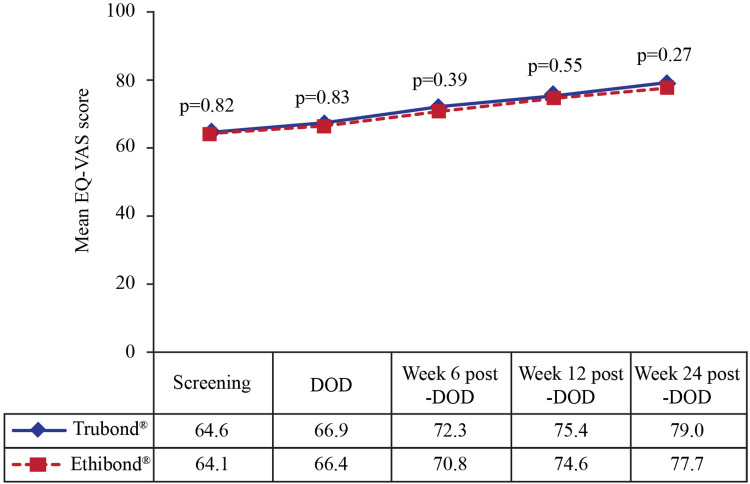
Global assessment of health by the EQ-VAS score in the Trubond® (n = 40) and Ethibond® (n = 42) groups. DOD: day of discharge; EQ-VAS: EuroQoL-Visual Analog Scale

However, significant differences (p < 0.05) regarding anxiety/depression on DOD (Figure 4e) and pain/discomfort at week six post-DOD (Figure 4d) were noted between the Trubond® and Ethibond® groups. Nevertheless, before the last follow-up, no pain/discomfort and anxiety/depression were achieved by 35 (87.5%) vs. 32 (76.2%) (p = 0.38) and 35 (87.5%) vs. 37 (88.1%) subjects, respectively, in the Trubond® vs. Ethibond® group. The mean duration at which they had no pain/discomfort and anxiety/depression was comparable between the groups (Table 2). Moreover, the mean values of the EQ-VAS score were 64.6 ± 10.8 and 64.1 ± 11.5 during screening in the Trubond® and Ethibond® groups, respectively, which gradually increased with each follow-up, and were 79.0 ± 5.00 and 77.7 ± 5.2, respectively at the last visit (Figure 5).

The non-serious adverse events recorded during the study period were cough (5.0%), general body pain and wound itching (2.5%), tiredness (2.5%), back pain and cough (2.5%), incision site pain (5.0%), and gastritis (2.5%) in the Trubond® group, whereas incision site pain (4.8%), gastritis (2.4%), general body pain and tiredness (2.4%), and general body pain (2.4%) in the Ethibond® group. The adverse events were mild in severity and not related to the study device. During the study, analgesics, antibiotics, and gastrointestinal medications were prescribed to the subjects, and the details of some of them are presented in Table 4.

**Table 4:** Concomitant or prescribed medications administered to the study participants.

Prescribed medications	Trubond^®^ (n = 40)	Ethibond^®^ (n = 42)
Analgesics, n (%)		
Paracetamol	28 (70.0)	32 (76.2)
Aceclofenac	6 (15.0)	3 (7.1)
Diclofenac	4 (10.0)	1 (2.4)
Antibiotics, n (%)		
Ceftriaxone	14 (35.0)	18 (42.9)
Cefuroxime	11 (27.5)	10 (23.8)
Gentamicin	12 (30.0)	15 (35.7)
Cefachlor	8 (20.0)	12 (28.6)
Gastrointestinal, n (%)		
Pantoprazole	24 (60.0)	25 (59.5)
Ranitidine	6 (15.0)	6 (14.3)

## Discussion

VHD is becoming common in the younger population of India, as more than 90% of the patients reported to have VHD were below 60 years of age. Combined MV and AV disease was observed in 34.5% of cases. The authors also observed that the disease of isolated MV was the most common (48.7%), while the involvement of AV constituted 16.2% [4]. In this study, the majority of subjects in the Trubond® group, who underwent AV or MV replacement, had severe stenosis combined with mild regurgitation. In the Ethibond® group, the common reason for AV replacement was severe aortic stenosis with mild aortic regurgitation, and for MV replacement, severe stenosis and moderate regurgitation, followed by only severe stenosis. Similar to a previously reported Indian study [4], MV was replaced in the majority of subjects in both suture groups of our study. To reduce the symptoms of valvular lesions and VHD-related complications and to prolong the patient’s survival, a surgical valve prosthesis is applied [17]. Successful valve replacement surgery is often reported to be associated with poor long-term prognosis, as 60.6% chances of survival with AV replacement (after 15 years) and 43.0% survival rates with MV replacement (after 20 years) have been recorded [1]. Incidence of PVL is a potentially serious complication that occurred in 2-10% and 7-17% of patients after AV and MV replacement, respectively [18]. Another study found 7.3% PVL after MV replacement surgery [19]. The PVL is a channel that is formed between the anatomical annulus and the prosthetic valve and directs the blood flow toward both the anterior and posterior direction [9], altering the cardiovascular hemodynamics. However, in this study, no incidence of PVL was observed in both suture groups at 26 weeks of index surgery.

Many risk factors are associated with the development of PVL, including annular calcification, tissue fragility, previous endocarditis, ongoing corticosteroid treatment along with the type of prosthesis used, and the surgical technique [20]. The type of prostheses is generally selected depending on the patient’s age, previous indication for anticoagulation use, as well as the preference of the patient and the surgeon [6]. A mechanical prosthesis is usually used in patients aged below 60 years and requires the use of lifelong anticoagulation, which can be eliminated with the use of bioprosthetics, but the risk of early infection and re-operation due to structural valve deterioration exists [6,21]. Mechanical valves are comparatively durable and calcification-resistant [21]. In our study, both mechanical and bioprosthesis valves were used, but mechanical tilting disc valves were used in the majority of subjects, followed by mechanical bileaflet valves. The use of pledgets is also expected to prevent PVL [12], and the application of pledget reinforcement in most subjects of the present study can be linked with no recorded incidence of PVL. The effectiveness of the interrupted suture technique for valve fixation is already established [11,12]. The use of the Trubond® or Ethibond® braided polyester suture in an interrupted manner for valvular prostheses fixation can be another reason for no PVL in the subjects of this study. The results relative to intraoperative handling characteristics of both sutures were similar and satisfactory. Non-suture-related adverse events of non-serious types further indicated the efficiency of both sutures and the surgery.

Generally, PVL is identified within the first year of valve replacement, and larger leakage may cause heart failure in 90% of patients [9]. A previous study reported 2% myocardial infarction and stroke after MV replacement [15]. Another study on MV replacement recorded 6.7% overall mortality due to infection (36.4%), cardiogenic shock (18.2%), and stroke (18.2%) [19]. According to data obtained from the Japanese multicenter registry, the incidence of cardiac and non-cardiac (due to infection) deaths was 26.6% and 22.5%, respectively [22]. A Sub-Saharan study from a low-middle-income region reported one-year survival for the entire cohort of 117 patients as 88.8 ± 2.1% [23]. Contrary to these findings, our study recorded no incidents of all-cause mortality, cardiac death, myocardial infarction, stroke, and superficial and deep incisional SSI at all follow-ups.

Low ventricular ejection fraction indicates left ventricle functional abnormality [24]. According to the American College of Cardiology, left ventricular systolic function is classified depending on ejection fraction as severe dysfunction (<30%), moderate dysfunction (30-39%), mild dysfunction (40-49%), normal (50-70%), and hyperdynamic (>70%) [25]. The recorded ejection fraction was 55.9 ± 6.2% and 56.4 ± 8.8% in the Trubond® and Ethibond® groups, respectively, at baseline which increased to 57.6 ± 4.2% and 57.7 ± 3.9%, respectively, after 26 weeks of surgery, suggesting normal left ventricular function and favorable outcome of valve replacement. The British Society of Echocardiography practical guidelines considered patients with a mean aortic gradient of 35-40 mmHg to have moderate-to-severe stenosis, and ≥40 mmHg as severe stenosis [26]. The current findings related to the mean aortic gradient indicated severe stenosis in subjects of both groups. However, after six months of AV replacement, the mean gradient had reduced. A previous study reported a similar reduction in mean AV gradient to 12.0 ± 11.9 and 16.8 ± 11.1 mmHg after 45 months of AV repair in patients who had preoperative peak aortic gradient of <20 and ≥20 mmHg, respectively [27]. In patients with rheumatic mitral stenosis, the mean transvalvular gradient was reported to be 7 mmHg in mild-to-moderate mitral stenosis and 14 mmHg in severe mitral stenosis, which was reduced to 4 and 6 mmHg, respectively, post-valvuloplasty [28]. At the final follow-up, the mean mitral transvalvular gradient of our study also decreased to ~4 mmHg in both groups. The results indicated successful valve replacement, resulting in unidirectional blood flow and diminished pressure across the chambers. The global incidence of mitral regurgitation is around 24.2 million which increased by 70% between 1990 and 2017, especially in developing nations; however, the prevalence of aortic regurgitation is highest in developed countries and is age-dependent [3]. Regurgitation increases the pressure of both left chambers and deteriorates the NYHA class [9]. In this study, both aortic and mitral regurgitations were present before surgery that improved with respective valve replacement, and only regurgitations of mild grade were noted in both groups at the end of the study. In addition, the regurgitations did not impact the NYHA class, as by the end of week 26, more than 70% of patients were classified as NYHA I, indicating improvement in functional ability. Moreover, the six-minute walk test, which is a well-tolerated and effective test to measure the functional capacity of patients [29], was also improved from the screening visit to week 24 post-DOD.

A recent retrospective cohort study on MV surgery reported re-hospitalization within one year of surgery in more than half (58.5%) of the patients [30]. On the contrary, in the present study, readmission was not recorded at any time point, and the lengths of ICU, as well as hospital stay, were comparable between the groups. Overall, the patients’ physiological and mental health and social functioning improved after the index surgery, as indicated by time to return to normal day-to-day activity and work, the five dimensions of EQ-5D, and the EQ-VAS score.

The study limitation was that the participants were blinded to the suture material used, but not the practitioner/s and the staff. Therefore, potential bias might have occurred in the assessment of the intraoperative suture handling characteristics. However, the comprehensive design and analysis of this study are its strengths, which authenticate the clinical use of the Trubond® suture in a wider range of populations. Moreover, the comparable results of both sutures propose the use of the Trubond® braided polyester suture for fixation of the valvular prosthesis along with all other surgeries indicated for the Ethibond® braided polyester suture.

## Conclusions

Valvular prosthesis fixation using the Trubond® or Ethibond® braided polyester suture resulted in no occurrence of PVL, which is a serious complication of valve replacement. The primary and secondary outcomes of this study demonstrated the clinical equivalence of the Trubond® and Ethibond® sutures. Therefore, in patients requiring AV or MV replacement, the Trubond® braided polyester suture can be used safely for the fixation of the valvular prosthesis.

## References

[1] Chen M, Yao X, Wang D (2021). Long-term cardiac remodeling associated with heart failure following left-ventricular valve replacement surgery: a retrospective study. Medicine (Baltimore).

[2] Yadgir S, Johnson CO, Aboyans V (2020). Global, regional, and mational burden of calcific aortic valve and degenerative mitral valve diseases, 1990-2017. Circulation.

[3] Aluru JS, Barsouk A, Saginala K, Rawla P, Barsouk A (2022). Valvular heart disease epidemiology. Med Sci (Basel).

[4] Sahu AK, Sagar P, Khanna R (2020). Etiology and distribution of isolated aortic stenosis in Indian patients - a study from a large tertiary care hospital in north India. Indian Heart J.

[5] Unger P, Clavel MA, Lindman BR, Mathieu P, Pibarot P (2016). Pathophysiology and management of multivalvular disease. Nat Rev Cardiol.

[6] Bernard J, Kalavrouziotis D, Marzouk M, Nader J, Bernier M, Pibarot P, Mohammadi S (2023). Prosthetic choice in mitral valve replacement for severe chronic ischemic mitral regurgitation: long-term follow-up. J Thorac Cardiovasc Surg.

[7] Playford D, Stewart S, Celermajer D (2020). Poor survival with impaired valvular hemodynamics after aortic valve replacement: the National Echo Database Australia study. J Am Soc Echocardiogr.

[8] Shah S, Alashi A, Pettersson GB (2019). Characteristics and longer-term outcomes of paravalvular leak after aortic and mitral valve surgery. J Thorac Cardiovasc Surg.

[9] Cruz-Gonzalez I, Antunez-Muiños P, Lopez-Tejero S, Sanchez PL (2022). Mitral paravalvular leak: clinical implications, diagnosis and management. J Clin Med.

[10] Chan PG, Chan EG, Seese L, Sultan I, Kilic A, Gleason TG, Chu D (2019). Safety and feasibility of a nonpledgeted suture technique for heart valve replacement. JAMA Surg.

[11] Azam H, Hussain G, Ahmed N, Raza Baig MA, Zaheer S, Ali Gilani SR (2015). Comparison of semi-continuous and interrupted suture techniques for mitral valve replacement. J Pak Med Assoc.

[12] Lee JO, Lee CH, Kim HJ (2020). Simple interrupted suturing for aortic valve replacement in patients with severe aortic stenosis. Korean J Thorac Cardiovasc Surg.

[13] Loona M, Bhushan R, chugh V, Jhajhria NS, Grover V, Gupta V (2020). Outcomes and safety of sternal closure using non-absorbable polyester braided suture: single tertiary care center experience of 5 years. Int J Sci Res.

[14] Kitamura T, Edwards J, Miyaji K (2017). Continuous suture technique for aortic valve replacement shortens cross-clamp and bypass times. Tex Heart Inst J.

[15] Salhiyyah K, Kattach H, Ashoub A (2017). Mitral valve replacement in severely calcified mitral valve annulus: a 10-year experience. Eur J Cardiothorac Surg.

[16] Beddermann C, Borst HG (1978). Comparison of two suture techniques and materials: relationship to perivalvular leaks after cardiac valve replacement. Cardiovasc Dis.

[17] Otto CM, Nishimura RA, Bonow RO (2021). 2020 ACC/AHA guideline for the management of patients with valvular heart disease: executive summary: a report of the American College of Cardiology/American Heart Association Joint Committee on Clinical Practice Guidelines. Circulation.

[18] Suh YJ, Hong GR, Han K (2016). Assessment of mitral paravalvular leakage after mitral valve replacement using cardiac computed tomography: comparison with surgical findings. Circ Cardiovasc Imaging.

[19] Moreira JL, Barletta PH, Baucia JA (2021). Morbidity and mortality in patients undergoing mitral valve replacement at a cardiovascular surgery referral service: a retrospective analysis. Braz J Cardiovasc Surg.

[20] Gürsoy MO, Güner A, Kalçık M, Bayam E, Özkan M (2020). A comprehensive review of the diagnosis and management of mitral paravalvular leakage. Anatol J Cardiol.

[21] Susak S, Velicki L, Popovi D, Burazor I (2013). Surgical valve replacement (bioprosthetic vs. mechanical). Calcific Aortic Valve Disease.

[22] Minamino-Muta E, Kato T, Morimoto T (2017). Causes of death in patients with severe aortic stenosis: an observational study. Sci Rep.

[23] Mve Mvondo C, Pugliese M, Ambassa JC, Giamberti A, Bovio E, Dailor E (2020). Mechanical heart valve replacement in a low-middle income region in the modern era: midterm results from a Sub-Saharan center. Thorac Cardiovasc Surg.

[24] Supomo S (2018). Prognostic factors in mitral valve replacement surgery at Dr. Sardjito General Hospital, Yogyakarta-Indonesia. Bali Med J.

[25] Kosaraju A, Goyal A, Grigorova Y, Makaryus AN (2023). Left Ventricular Ejection Fraction. https://pubmed.ncbi.nlm.nih.gov/29083812/.

[26] Ring L, Shah BN, Bhattacharyya S (2021). Echocardiographic assessment of aortic stenosis: a practical guideline from the British Society of Echocardiography. Echo Res Pract.

[27] Vohra HA, Whistance RN, de Kerchove L, Glineur D, Noirhomme P, El Khoury G (2013). Influence of higher valve gradient on long-term outcome after aortic valve repair. Ann Cardiothorac Surg.

[28] El Sabbagh A, Reddy YN, Barros-Gomes S (2019). Low-gradient severe mitral stenosis: hemodynamic profiles, clinical characteristics, and outcomes. J Am Heart Assoc.

[29] Giannitsi S, Bougiakli M, Bechlioulis A, Kotsia A, Michalis LK, Naka KK (2019). 6-minute walking test: a useful tool in the management of heart failure patients. Ther Adv Cardiovasc Dis.

[30] Havers-Borgersen E, Butt JH, Strange J, Carranza CL, Køber L, Fosbøl EL (2023). Mortality and rehospitalization after mitral valve surgery as a function of age and key comorbidities. Am Heart J.

